# Therapeutic mechanisms of umbilical cord mesenchymal stem cell–derived exosomes in ischemic stroke: A transcriptomic and metabolomic study

**DOI:** 10.4103/NRR.NRR-D-24-01219

**Published:** 2025-08-13

**Authors:** Baoxi Shen, Jing Chen, Ning Liu, Jingyi Hou, Yiwu Dai

**Affiliations:** 1 Medical School of Chinese PLA, Beijing, China; 2 Department of Neurosurgery, the First Medical Centre, Chinese PLA General Hospital, Beijing, China; 3 Physical Examination Center, The People’s Hospital of Pizhou, Xuzhou, Jiangsu Province, China; 4 Department of Neurosurgery, the Seventh Medical Center, Chinese PLA General Hospital, Beijing, China; 5 Beijing Key Laboratory of Traditional Chinese Medicine Basic Research on Prevention and Treatment for Major Diseases, Experimental Research Center, China Academy of Chinese Medical Sciences, Beijing, China

**Keywords:** exosomes, ischemic stroke, mesenchymal stem cells, metabolomics, middle cerebral artery occlusion, stroke, transcriptomics

## Abstract

Ischemic stroke remains a leading cause of disability and death, with mesenchymal stem cell–derived exosomes emerging as a promising therapeutic avenue. However, the optimal timing and underlying therapeutic mechanisms of exosome treatment require further elucidation. In this study, we used a murine model of middle cerebral artery occlusion to investigate the therapeutic efficacy of human umbilical cord mesenchymal stem cell–derived exosomes administered intravenously at an early (6 hours) or delayed (3 days) time point post-ischemia. Compared with delayed treatment, early administration of exosomes resulted in significantly superior efficacy, as evidenced by improved neurological function scores and reduced infarct volumes. Transcriptomic analysis of brain tissues from mice receiving early exosome treatment revealed marked downregulation of inflammation-related genes, including *Ccl2*, *Ccl5*, *Cxcl10*, *Il-1*β, *Il-6*, *Itgam*, *Itgax*, and *Tnf-α*. Metabolomic profiling of these brain tissues further identified modulation of key metabolites, including trimethylamine N-oxide, glutathione, 1-stearoyl-rac-glycerol, and phosphatidylcholine, suggesting that alteration of metabolic pathways contributes to the therapeutic effect. Integrated transcriptomic and metabolomic analysis pinpointed significant modulation of pathways involving metabolism of eicosapentaenoic acid, lysine, propanoate, and tyrosine. These findings suggest that umbilical cord mesenchymal stem cell–derived exosomes, particularly when administered early post-ischemia, exert their neuroprotective effects by broadly suppressing inflammatory pathways and modulating key metabolic processes in the ischemic brain, highlighting their potential as a therapeutic intervention for ischemic stroke.

## Graphical abstract

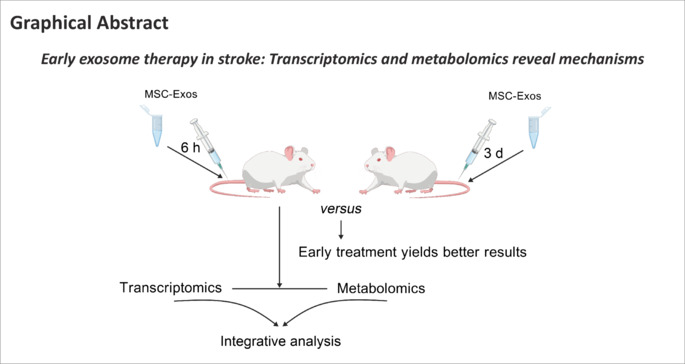

## Introduction

Globally, stroke is one of the leading causes of motor, sensory, and cognitive impairments, and even death, among patients. Ischemic stroke, which is typically caused by a blood clot, accounts for approximately 70% of all strokes (Herpich and Rincon, 2020; Walter 2022). Apart from its severe impact on individuals, ischemic stroke imposes a significant economic burden on global healthcare resources (Bhatia et al., 2018). Preclinical studies have demonstrated the efficacy of mesenchymal stem cells (MSCs) in treating ischemic stroke in animal models, delivering significant improvements in neurological function and reduced infarct volume (Li et al., 2023; Behzadifard et al., 2023; Tian et al., 2024). While the clinical translation of MSC therapy faces challenges, including low cell engraftment and potential immunogenicity, MSC-derived exosomes offer a promising alternative that circumvents these limitations. Exosomes, as naturally secreted nanovesicles, are inherently biocompatible, readily crossing the blood–brain barrier to allow targeted delivery of therapeutic molecules to the ischemic brain. A series of animal experiments testing the treatment of ischemic stroke with MSC-derived exosomes all achieved good results (Kim et al., 2020; Jiang et al., 2023; Liu et al. 2023b; Pan et al., 2023). Multiple studies support the utility of exosomes as intercellular messengers, acting as efficient carriers of biomaterials that play an indispensable role in brain homeostasis, including nucleic acids, proteins, and metabolites (Arya et al., 2024; Cunha E Rocha et al., 2024; Yin et al., 2025). In recent years, increasing research has shown that exosomes exist stably in circulation and are capable of penetrating the blood–brain barrier (Williams et al., 2020; Venkat et al. 2020). These characteristics underscore the therapeutic properties of exosomes in the management of ischemic stroke. However, several unresolved issues must be addressed before translating MSC-derived exosome research from animal experiments to clinical applications. The optimal timing for exosome therapy derived from MSCs following ischemic stroke remains a crucial question that warrants further investigation. Should it be performed early or delayed? Additionally, the mechanisms underlying MSC-derived exosome therapy for ischemic stroke are not fully understood.

Here, we aimed to explore the optimal timing and therapeutic mechanisms of umbilical cord-MSC (UC-MSCs)-derived exosome treatment for ischemic stroke in mice, evaluating therapeutic efficacy following intravenous tail vein injections of exosomes at 6 hours or 3 days post-middle cerebral artery occlusion (MCAO). Therapeutic effects were assessed using behavioral tests and staining with 2,3,5-triphenyltetrazolium chloride (TTC), hematoxylin and eosin (HE), and Nissl. To investigate the mechanisms underlying the disparate therapeutic outcomes observed across different treatment time points, we selected the optimal time point for in-depth transcriptomic and metabolomic studies.

## Methods

### Isolation and characterization of human umbilical cord mesenchymal stem cell–derived exosomes

#### Isolation of umbilical cord mesenchymal stem cell–derived exosomes

Umbilical cord samples were collected from healthy neonates delivered via cesarean section in the Department of Obstetrics and Gynecology at the General Hospital of the People’s Liberation Army. Written informed consent was secured from all participating mothers. The use of UC-MSCs was approved by the Ethics Committee of the General Hospital of the People’s Liberation Army on November 17, 2017 (approval No. 2017-79). The human UC-MSCs were cultured to **~**85% confluence. The culture medium (Cat# 6114011, Dakewe Co., Ltd., Shenzhen, China) was discarded, and the cells were washed thrice with sterile Dulbecco’s phosphate-buffered saline (DPBS; Thermo Fisher Scientific, Waltham, MA, USA) before adding serum-free culture medium (Cat# 6114011) for another 48 hours of culture. The cell supernatant was collected and transferred to a 50-mL centrifuge tube. After centrifugation at 500 × *g* for 5 minutes at 4°C, the supernatant was collected and transferred to a new 50-mL centrifuge tube. Centrifugation at 2000 × *g* for 30 minutes at 4°C was performed, followed by another centrifugation step at 10,000 × *g* for 60 minutes. The supernatant was then passed through a 0.22-μm sterile filter and transferred to an ultra-high-speed centrifuge tube. Following ultracentrifugation at 120,000 × *g* for 1 hour at 4°C, the supernatant was discarded, and the resulting exosome pellet was resuspended in 200 μL of sterile DPBS.

#### Identification of umbilical cord mesenchymal stem cells

Isolated and cultured UC-MSCs were characterized using an MSC surface marker detection kit (Cat# 562245, BD Biosciences, San Jose, CA, USA). Three Eppendorf tubes were used for staining with antibodies against CD90, CD105, and CD73, and a fourth tube served as a negative control, stained with the human MSC negative cocktail of antibodies against CD34, CD11b, CD19, CD45, and HLA-DR. Log-phase UC-MSCs were detached using 0.25% trypsin–0.01% EDTA. Following a wash with DPBS, the four tubes were centrifuged at 750 × *g* for 5 minutes, and the supernatant was discarded. The appropriate antibody was added to each tube, the cells were incubated at 4°C for 30 minutes in the dark, washed with 500 μL DPBS, and centrifuged at 750 × *g* for 5 minutes. After discarding the supernatant and repeating the washing step one time, the cells were resuspended in 500 μL DPBS. Finally, cell surface markers were detected using a C6 flow cytometer (Becton, Dickinson and Company, San Jose, CA, USA).

#### Exosome characterization

For particle size determination, exosomes were diluted 40-fold with sterile DPBS and passed through a 0.22-μm sterile filter and nanoparticle tracking analysis (NTA) was performed with the ResunTech NanoCoulter G (ResunTech, Shenzhen, China). For transmission electron microscopy (TEM), exosomes were washed thrice with 1 mL DPBS. A 0.5-mL solution of 2% osmium tetroxide was added for fixation at 4°C for 2 hours. After washing with 1 mL DPBS, the samples were dehydrated using a graded series of ethanol solutions (50%, 70%, 80%, 90%, and 100%) for 15 minutes each, followed by two 20-minute incubations in 100% ethanol. Acetone was used for two 15-minute washes to remove the ethanol. The samples were then embedded in resin, polymerized at 65°C for 48 hours, stained with uranyl acetate and lead acetate for 10 minutes each, and washed. Exosome morphology was observed using an HT7800 transmission electron microscope (Hitachi, Tokyo, Japan). For western blot analysis, proteins were separated by electrophoresis through 15% polyacrylamide gels and transferred to 0.22-μm polyvinylidene fluoride membranes (Millipore, Burlington, MA, USA) at 275 mA for 85 minutes. Membranes were blocked with 1% bovine serum albumin (BSA; Beyotime, Shanghai, China) in Tris-buffered saline with Tween 20 (TBST; Abcam, Cambridge, UK) for 1 hour at room temperature. The following primary antibodies were diluted in 1% BSA-TBST and incubated overnight at 4°C: rabbit anti-CD9 (1:5000 dilution, Cat# 13403, Cell Signaling Technology, Danvers, MA, USA), rabbit anti-tumor susceptibility gene 101 (TSG101; 1:1000, Cat# ab125011, Abcam), mouse anti-heat shock protein 70 (HSP70; 1:4000, Cat# 66183-1-Ig, ProteinTech, Rosemont, IL, USA), and rabbit anti-Calnexin (1:5000, Cat# ab133615, Abcam). After three TBST washes (10 minutes each), membranes were incubated with the following secondary antibodies in 1% BSA-TBST for 1 hour at room temperature (20–25°C): horseradish peroxidase-conjugated anti-rabbit IgG (1:5000, Cat# 7074, Cell Signaling Technology) or anti-mouse IgG (1:2000, Cat# 7076S, Cell Signaling Technology). Membranes were washed three times with TBST (10 minutes each) and visualized using chemiluminescence.

### Animals and study groups

Male C57BL/6J mice (age: 8 weeks; bodyweight: 22–24 g) were obtained from Beijing Huafukang Company (license No. SCXK (Jing) 2019-0008). The experimental procedures were conducted in accordance with the guidelines and protocols approved by the Ethics Committee of the Seventh Medical Center of the People’s Liberation Army General Hospital (approval No. 2024-9; approval date: February 19, 2024). All experiments were designed and reported in accordance with the Animal Research: Reporting of *In Vivo* Experiments (ARRIVE) guidelines (Percie du Sert et al., 2020). Experimental animals were housed in a clean environment, with temperatures maintained at 15–30°C and humidity of 40%–70%, and a 12-hour light/12-hour dark cycle (uniform light intensity maintained within 15–20 lx during the light phase). To investigate the optimal timing of MSC-derived exosome therapy for ischemic stroke, mice were randomly assigned to four groups: Sham group (*n* = 10), which underwent a sham surgery involving exposure of the internal carotid artery but no thread embolism; MCAO group (*n* = 20), which received MCAO without any subsequent treatment; and early and late treatment groups (*n* = 20 each), which received 100 µg of MSC-derived exosomes (dissolved in 200 µL DPBS) by intravenous tail vein injection at 6 hours (Exo-6h) or 3 days (Exo-3d) post-MCAO induction, respectively. Seven days after treatment, behavioral assessments were performed, followed by brain tissue collection for TTC, HE, and Nissl staining.

Comprehensive transcriptomic and metabolomic analyses were conducted on tissues from the early time point, which demonstrated superior therapeutic efficacy, in three experimental groups: Sham group (*n* = 6), which underwent the sham surgery described above; DPBS group (*n* = 10), which received a 200-μL intravenous tail vein injection of DPBS solution at 6 hours post-MCAO modeling; and Exo group (*n* = 10), which received 100 μg of MSC-derived exosomes (dissolved in 200 μL DPBS) by intravenous tail vein injection at 6 hours post-MCAO modeling. Brain tissues were collected at 7 days post-surgery for transcriptomic and metabolomic studies.

### Establishment of the cerebral ischemia model

MCAO was performed following a previously outlined procedure (Li and Zhang, 2021). Mice were anesthetized by inhalation of 3% isoflurane (RWD Life Science, Shenzhen, China) with an oxygen flow rate of 0.3 L/min in an induction chamber. Once adequate anesthesia was achieved, the mice were transferred to a nose cone connected to a gas anesthesia machine (RWD Life Science, Shenzhen, China) and maintained on 1.5% isoflurane. During the procedure, body temperature was maintained using a heated pad, and limbs were secured with medical tape. The neck skin was disinfected with iodine solution, followed by a surgical incision. The external and internal carotid arteries were ligated sequentially, and a transverse incision was made between the ligatures of the right external carotid artery. For occlusion, a thread (Cat# 6022, Doccol Corporation, Sharon, MA, USA) was inserted into the incision to a depth of approximately 1 cm for 90 minutes, after which the thread was slowly withdrawn. The vessels were then treated while the wound was sutured and local area disinfected, after which the mice were returned to their cages for observation during recovery.

### Neurological function impairment scoring

Mice were assessed for neurological deficits using the modified neurological severity score (mNSS) (Qian et al., 2024). The total possible score is 18, with higher scores indicating more severe neurological damage.

### Behavioral evaluation

Behavioral assessment was conducted using a cylinder test (Shi et al., 2021). Mice were observed in a standardized cylindrical container made of organic glass and the movement of the forelimbs was measured. The cylinder diameter (15 cm) and height (30 cm) were designed to ensure consistency and repeatability of the experimental environment. Under normal physiological conditions, mice exhibit exploratory behavior inside the cylinder, attempting to stand upright and naturally using their forepaws to touch the cylinder wall. To quantify this behavior, we established a 15-minute observation period and recorded in detail the frequency of contacts made by the left forelimb, right forelimb, and both forelimbs with the cylinder wall. These data provided us with objective indicators of forelimb usage in the groups.

### 2,3,5-Triphenyltetrazolium chloride staining

Following deep anesthesia as described above, intact mouse brains were immediately excised, frozen at –20°C for 20 minutes, and coronally sectioned into 2-mm thick slices. The slices were then incubated in TTC solution (Solarbio, Beijing, China) for 15 minutes, protected from ambient light. Cerebral infarct volume was subsequently quantified using ImageJ software (NIH, Bethesda, MD, USA). The infarct volume was expressed as a percentage of the total brain area.

### Histopathological staining

Mouse brain tissues were fixed in 4% paraformaldehyde (Beyotime) for 24 hours, dehydrated, embedded in paraffin, and sliced into 5 μm-thick sections. The sections were stained with HE Solution (Cat# G1120, Solarbio) or Nissl Solution (Cat# G1430, Solarbio) for histopathological assessment of neuronal damage on an OLYMPUS CX23 microscope (Olympus, Tokyo, Japan).

### Metabolomics analysis

#### Metabolite extraction

Mouse brain tissue metabolites were extracted from 80 mg of tissue using 1 mL of a cold solvent mixture (methanol/acetonitrile/H_2_O, 2:2:1 v/v/v). Samples were adequately vortexed, homogenized by MP homogenizer (24 × 2, 6.0 M/S, 60 seconds, twice), and sonicated at 4°C (30 minutes each time, twice). The resulting lysate was then centrifuged (14,000 × *g*, 20 minutes, 4°C). Subsequently, the resulting supernatant was dried in a vacuum centrifuge at 4°C. For liquid chromatography (LC)-mass spectrometry (MS) analysis, the dried samples were redissolved in 100 μL of acetonitrile/water (1:1 v/v).

#### Liquid chromatography-mass spectrometry analysis

For untargeted polar metabolomics, we utilized the Sciex TripleTOF 6600 mass spectrometer (SCIEX, Framingham, MA, USA), coupled with hydrophilic interaction chromatography through electrospray ionization at Shanghai Applied Protein Technology Co., Ltd. For LC separation, an ACQUIY UPLC BEH Amide column (2.1 mm × 100 mm, 1.7 µm particle size) (Waters Corporation, Milford, MA, USA) was employed, with a gradient of solvent A (25 mM ammonium acetate and 25 mM ammonium hydroxide in water) and solvent B (acetonitrile). Operating conditions included a flow rate of 0.4 mL/minute, column temperature of 25°C, autosampler temperature of 5°C, and an injection volume of 2 µL. The mass spectrometer operated in negative and positive ionization modes, utilizing specified ESI source parameters. MS acquisition spanned the m/z range of 60–1000 Da, with a time-of-flight MS scan accumulation time of 0.20 s/spectra.

#### Data analysis

Raw MS data were converted to MzXML format using ProteoWizard MSConvert (ISB, Seattle, WA, USA) and subsequently analyzed using XCMS software (Scripps Research Institute, La Jolla, CA, USA). Peak picking parameters included centWave m/z = 25 ppm, peakwidth = c (10, 60), and prefilter = c (10, 100). Peak grouping parameters were bw = 5, mzwid = 0.025, and minfrac = 0.5. Metabolites were identified using MS/MS spectra against an in-house database. Processed data, normalized to total peak intensity, underwent analysis in SIMCA-P (version 14.1, Umetrics, Umea, Sweden) using orthogonal partial least squares discriminant analysis (OPLS-DA) with permutation testing (100 permutations) to prevent overfitting. The following criteria were employed to identify differential metabolites (DMs): variable importance projection (VIP) score > 1, Log|fold change (FC)| > 1, and *p* value < 0.05. MetaboAnalyst (https://www.metaboanalyst.ca/, version 6.0) was used to identify associated metabolic pathways.

### Transcriptomics analysis

#### RNA extraction

Total RNA was extracted from mouse brain samples using TRIzol Reagent (Thermo Fisher Scientific). RNA quality was assessed by measuring the A260:A280 ratio using a Nanodrop ND-2000 (Thermo Fisher Scientific) and the RNA integrity number (RIN) was determined using an Agilent Bioanalyzer 4150 (Agilent Technologies, Santa Clara, CA, USA). Only samples meeting quality criteria were used for library construction.

#### Library preparation and sequencing

Paired-end libraries were prepared using an ABclonal mRNA-seq Lib Prep Kit (ABclonal, Wuhan, China), in accordance with the manufacturer’s instructions. Purification of mRNA from 1 μg total RNA was performed using oligo (dT) magnetic beads. For cDNA synthesis, we used random hexamer primers and reverse transcriptase for first-strand cDNA, and DNA polymerase I for second-strand cDNA, with reactions containing RNAse H, buffer, and dNTPs. Adapter-ligated cDNA fragments underwent polymerase chain reaction (PCR) amplification, purification on an AMPure XP system (Beckman Coulter, Brea, CA, USA), and library quality assessment on an Agilent Bioanalyzer 4150. Sequencing was conducted on an Illumina Novaseq 6000 (Illumina, San Diego, CA, USA) with 150-bp paired-end reads.

#### Data analysis

Differential expression analysis was conducted using the “DESeq2” package in R (Bioconductor, Seattle, WA, USA), identifying differentially expressed genes (DEGs) based on the criteria of |log2(FC)| > 1 and a *P* value < 0.05. Gene Ontology (GO) enrichment and Kyoto Encyclopedia of Genes and Genomes (KEGG) pathway analyses of DEGs between the DPBS *versus* Sham and Exo versus DPBS groups were conducted using DAVID (https://david.ncifcrf.gov/) and Metascape (http://metascape.org). The protein–protein interaction network (PPI) of reversed DEGs was constructed using the STRING database (https://cn.string-db.org/) (Szklarczyk et al., 2021) and visualized in Cytoscape 3.7.2 (Cytoscape Consortium, San Francisco, CA, USA). Topological parameters were calculated using the cytoHubba plug-in, highlighting nodes with high degrees as key targets (Hou et al., 2021).

### Integrated metabolomics and transcriptomics analysis

Identification and analysis of critical metabolic pathways were performed using the Metscape plug-in within Cytoscape 3.7.2, utilizing the pathway-based mode (Gao et al., 2010) to integrate DMs from the metabolomics dataset and DEGs from the transcriptomics dataset. To enhance our understanding of the potential therapeutic efficacy of MSC-derived exosomes in MCAO treatment, we constructed a network comprising compounds, reactions, enzymes, and genes associated with key metabolites and DEGs involved in these metabolic pathways. This network was visualized within Cytoscape 3.7.2, enabling a comprehensive analysis of the integrated data.

### Quantitative reverse transcription-polymerase chain reaction

Total RNA was extracted from mouse brain samples using TRIzol Reagent, following the manufacturer’s instructions, then reverse transcribed to cDNA using the SuperRT III All-in-one RT Mix with gDNA Remover kit (Biosharp, Hefei, China). The expression levels of targeted mRNAs were then detected using the SYBR Green qPCR Mix kit (Biosharp). The gene-specific primer sequences are shown in **Table 1**. Relative mRNA expression was normalized to the β*-actin* gene and calculated using the 2^–ΔΔCt^ method.

**Table 1:** Sequences of primers used for quantitative reverse transcription-polymerase chain reaction

Gene	Sequence (5'–3')
*Tnf-α*	F: CTG AAC TTC GGG GTG ATC GG
	R: GGC TTG TCA CTC GAA TTT TGA GA
*Il-6*	F: CTG CAA GAG ACT TCC ATC CAG
	R: AGT GGT ATA GAC AGG TCT GTT GG
*Cd4*	F: CTT CGC AGT TTG ATC GTT TTG AT
	R: CCG GAC TGA AGG TCA CTT TGA
*Il-1β*	F: TTC AGG CAG GCA GTA TCA CTC
	R: GAA GGT CCA CGG GAA AGA CAC
*Itgam*	F: CCA TGA CCT TCC AAG AGA ATG C
	R: ACC GGC TTG TGC TGT AGT C
*Ccl2*	F: TAA AAA CCT GGA TCG GAA CCA AA
	R: GCA TTA GCT TCA GAT TTA CGG GT
*Ccl5*	F: GCT GCT TTG CCT ACC TCT CC
	R: TCG AGT GAC AAA CAC GAC TGC
*Cxcr4*	F: CTT CTG GGC AGT TGA TGC CAT
	R: CTG TTG GTG GCG TGG ACA AT
*Itgb2*	F: CAG GAA TGC ACC AAG TAC AAA GT
	R: GTC ACA GCG CAA GGA GTC A
*Cxcl10*	F: CCA AGT GCT GCC GTC ATT TTC
	R: GGC TCG CAG GGA TGA TTT CAA
*Ccr5*	F: ATG GAT TTT CAA GGG TCA GTT CC
	R: CTG AGC CGC AAT TTG TTT CAC
*Tgfb1*	F: CCA CCT GCA AGA CCA TCG AC
	R: CTG GCG AGC CTT AGT TTG GAC
*Itgax*	F: GTG CTG AGT TCG GAC ACA GT
	R: AGA GGC CAC CTA TTT GGT TAG T
*Ccr1*	F: TGG GTG AAC GGT TCT GGA AG
	R: GGT CCT TTC TAG TTG GTC CAC A
*Ccl11*	F: GAA TCA CCA ACA ACA GAT GCA C
	R: ATC CTG GAC CCA CTT CTT CTT
*Ccl4*	F: TTC CTG CTG TTT CTC TTA CAC CT
	R: CTG TCT GCC TCT TTT GGT CAG
*Ccl3*	F: TGT ACC ATG ACA CTC TGC AAC
	R: CAA CGA TGA ATT GGC GTG GAA
*Ccl12*	F: TGC ATC AGT GAC GGT AAA CCA
	R: TTC TTC AGC CGT GCA ACA ATC
*Cxcl16*	F: TTA TCA GGT TCC AGT TGC AGT C
	R: TGG TGG TGA AAA CTC TTC CCA
*β-actin*	F: AGG AGT ACG ATG AGT CCG GC
	R: AAA ACG CAG CTC AGT AAC AGT C

Ccl11: Chemokine (C–C motif) ligand 11; Ccl12: chemokine (C–C motif) ligand 12; Ccl2: C–C motif chemokine ligand 2; Ccl3: chemokine (C–C motif) ligand 3; Ccl4: chemokine (C–C motif) ligand 4; Ccl5: chemokine (C–C motif) ligand 5; Ccr1: C–C chemokine receptor type 1; Ccr5: C–C chemokine receptor 5; Cd4: T-cell surface glycoprotein CD4; Cxcl10: C–X–C motif chemokine ligand 10; Cxcl16: C–X–C motif chemokine ligand 16; Cxcr4: C–X–C chemokine receptor type 4; F: forward; Il-1β: interleukin-1β; Il-6: interleukin-6; Itgam: integrin subunit alpha M; Itgax: integrin subunit alpha X; Itgb2: integrin beta-2; R: reverse; Tgfb1: transforming growth factor beta 1; Tnf-α: tumor necrosis factor-α.

### Statistical analysis

Sample sizes were not predetermined using statistical methods, but are similar to those reported in previous publications (Li et al., 2023). Statistical analysis was conducted using SPSS 21.0 (IBM Corp., Armonk, NY, USA), with the results expressed as means ± standard error of the mean (SEM). Group comparisons were performed through one-way analysis of variance with Tukey’s *post hoc* test. A *P* value < 0.05 was considered statistically significant.

## Results

### Early mesenchymal stem cell-exosome administration is therapeutically superior to late treatment

**Figure 1:**
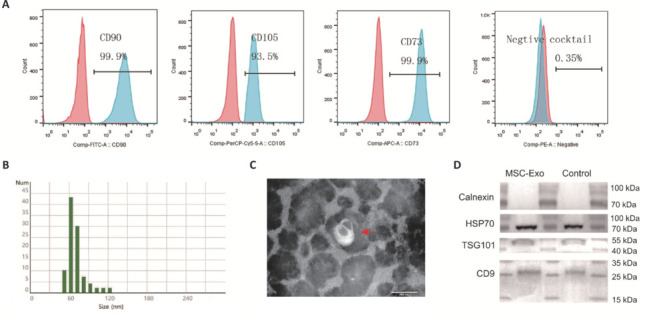
Characterization of umbilical cord-*derived* mesenchymal stem cells (UC-MSCs) and their exosomes. (A) Identification of uc-MSCs based on positive expression of CD90, CD105, and CD73, and negative expression of CD34, CD11b, CD19, CD45, and HLA-DR. (B) Nanoparticle tracking analysis of the size distribution of exosomes. (C) Transmission electron microscopy visualization of the morphology of exosomes (red arrow). Scale bar: 100 nm. (D) Western blot detection of CD9, TSG101, and HSP70, and the absence of calnexin, confirming successful exosome isolation. MSC-Exo: Exosomes isolated from the supernatant of fifth-generation UC-MSCs; Control: exosome standard.

Flow cytometry analysis of the UC-MSCs used in this study revealed a surface marker profile consistent with MSC identity: high expression of CD90, CD105, and CD73, and minimal expression of CD34, CD11b, CD19, CD45, and HLA-DR (**Figure 1A**). To comprehensively analyze the isolated exosomes, we employed NTA, TEM, and western blotting. NTA showed an exosome size distribution with a mean diameter of 67 nm (standard deviation, 13.9 nm), primarily ranging from 51 to 123 nm, and a median diameter of 64 nm (**Figure 1B**). TEM revealed nano-sized vesicles with diameters ranging from **~**30 to 150 nm and a characteristic flattened spheroid morphology (**Figure 1C**). Western blot analysis, employed to assess the purity of isolated exosomes, confirmed the presence of exosomal markers CD9, TSG101, and HSP70. The absence of calnexin, an endoplasmic reticulum marker, further indicated the purity of the isolated exosomes (**Figure 1D**).

**Figure 2:**
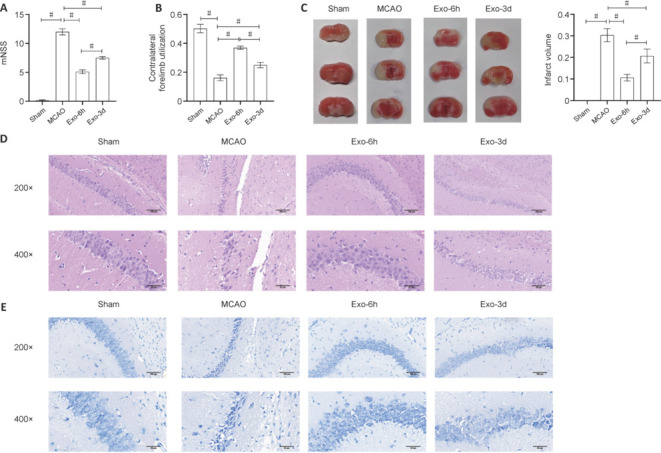
Assessment of therapeutic efficacy in different treatment groups of mice. (A) Modified neurological severity score (mNSS) in each group (*n* = 10). (B) Cylinder test of contralateral forelimb utilization in each group (*n* = 10). (C) Representative 2,3,5-triphenyltetrazolium chloride (TTC)-stained brain sections and quantitative analysis of infarct volume in each group (*n* = 3). White regions indicate the infarct area. (D) Representative hematoxylin and eosin-stained brain sections in each group (*n* = 3). Scale bars: 50 μm. (E) Representative Nissl-stained brain sections in each group (*n* = 3). Scale bars: 100 μm. Data in A–C are expressed as mean ± SEM. #*P* < 0.05 (one-way analysis of variance with Tukey’s *post hoc* test). Exo: Exosome; MCAO: Middle cerebral artery occlusion.

To determine the optimal therapeutic window for UC-MSC-derived exosome administration, we evaluated the effects of early (6 hours) *versus* late (3 days) treatment on neurological function, infarct volume, and hippocampal histopathology in a murine model of ischemic stroke. We found that the early (Exo-6h) and late (Exo-3d) treatment groups showed significantly lower mNSS scores than the untreated model (MCAO) group (*P* < 0.05; **Figure 2A**). Furthermore, the mNSS score was significantly lower in the Exo-6h group than in the Exo-3d group (*P* < 0.05; **Figure 2A**). To assess post-stroke asymmetric limb usage, we conducted a cylinder test. At 7 days post-treatment, both the Exo-6h and Exo-3d groups showed significant improvement in the usage of the contralateral limb compared with the MCAO group (*P* < 0.05; **Figure 2B**), with a significantly greater effect in the Exo-6h group than in the Exo-3d group (*P* < 0.05, **Figure 2B**).

TTC staining of brain tissues showed that, compared with the MCAO group, both the Exo-6h and Exo-3d groups exhibited significant reductions in infarct volume (*P* < 0.05), with a significantly smaller post-ischemia infarct volume in the Exo-6h group than in the Exo-3d group (*P* < 0.05; **Figure 2C**). HE staining revealed distinct differences in hippocampal neuron morphology across the groups (**Figure 2D**). Neurons in the Sham group displayed intact morphology and orderly arrangement, indicating normal physiological status. By contrast, the MCAO group exhibited significant neuronal injury characterized by degeneration and necrosis. The Exo-6h group showed largely intact neurons with relatively regular morphology and occasional cellular lysis. The Exo-3d group exhibited less severe neuronal injury than the MCAO group and, while most neurons retained a regular arrangement and normal morphology, some pyknotic nuclei were observed. Nissl staining also showed notable differences among the groups (**Figure 2E**). The Sham group exhibited neatly arranged neurons with abundant Nissl substance. The MCAO group showed disrupted neuronal arrangement, edema, necrosis, and pale Nissl staining. In the Exo-6h group, the Nissl substance showed a relatively orderly arrangement with occasional neuronal edema. The Exo-3d group displayed largely well-organized and clearly visible Nissl substance, with edema and necrosis of a few neurons. Taken together, these multifaceted assessments unequivocally demonstrated the superior neuroprotective efficacy of early exosome administration compared with delayed treatment in the context of ischemic stroke.

### Omics analyses of mesenchymal stem cell-derived exosome therapy delivered at 6 hours post-ischemia

#### Differentially expressed gene and protein–protein interaction network analyses

**Figure 3:**
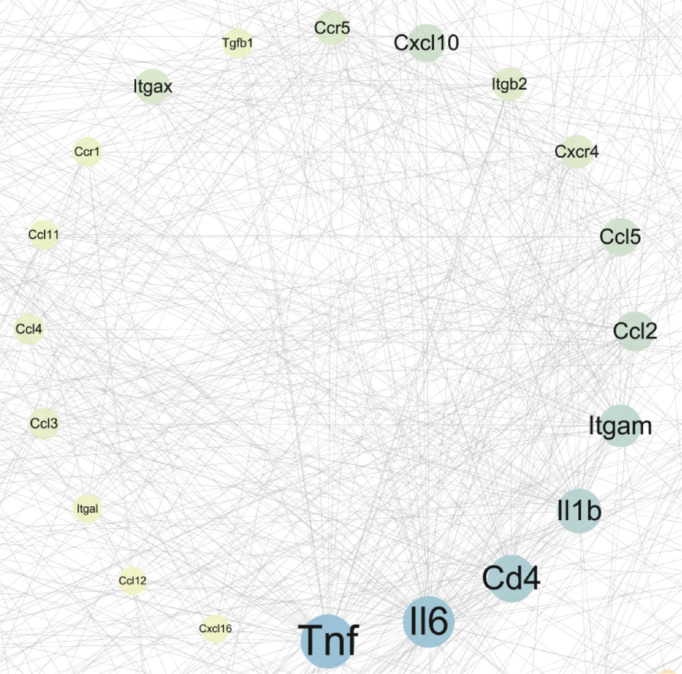
Protein–protein interaction network analysis of differentially expressed proteins in the DPBS–Sham and Exo–DPBS group comparisons. Proteins encoded by differentially expressed genes with significant alterations in both the DPBS–Sham and Exo–DPBS comparisons were incorporated into the network. Nodes within the network represent individual proteins, with the size of each node proportional to its degree of connectivity. The lines connecting nodes signify predicted functional relationships between the connected proteins. DEGs: Differentially expressed genes; DPBS: Dulbecco’s phosphate-buffered saline; Exo: exosome.

To elucidate the potential mechanisms underlying the exosome-mediated improvement in neural injury in MCAO mice, we analyzed the DEGs identified in the DPBS–Sham and Exo–DPBS group comparisons using the STRING online database. Subsequently, we constructed a visual PPI network comprising 486 nodes and 1181 edges (interactions). Assessment of the topological characteristics of the PPI network led to the identification of the following major targets: tumor necrosis factor-α (Tnf-α), interleukin (Il)-6), T-cell surface glycoprotein CD4 (Cd4), Il-1β, integrin subunit alpha M (Itgam), C–C motif chemokine ligand (Ccl)2, Ccl5, C–X–C chemokine receptor type 4 (Cxcr4), integrin beta-2 (Itgb2), C–X–C motif chemokine ligand (Cxcl)10, Ccr5 (chemokine (C–C motif) ligand 5), transforming growth factor beta 1 (Tgfb1), integrin subunit alpha X (Itgax), C–C chemokine receptor type 1 (Ccr1), Ccl11, Ccl4, Ccl3, Ccl12, and Cxcl16 (**Figure 3**). We hypothesize that these proteins serve as key targets for the therapeutic effects of MSC-derived exosomes in ischemic stroke.

### GO functional enrichment analysis of differentially expressed genes

**Figure 4:**
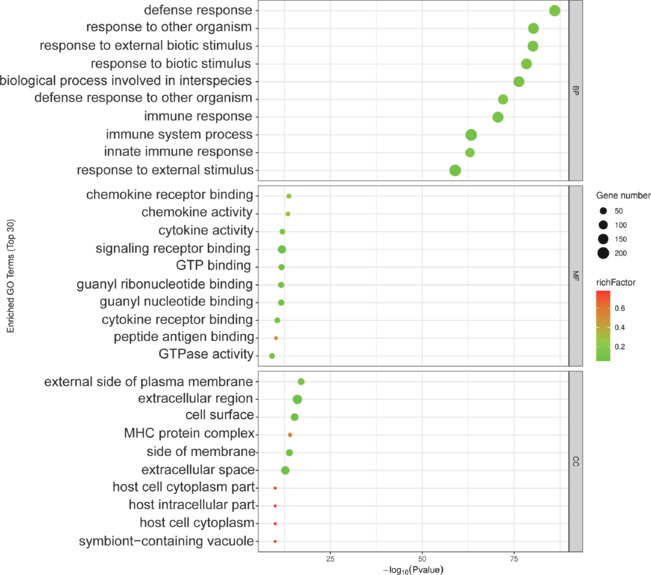
Gene Ontology (GO) enrichment of differentially expressed genes in the Exo *vs*. DPBS group comparison. GO terms, listed on the y-axis, are categorized by biological process, molecular function, and cellular component. The x-axis represents the –log10(*P* value). Point size corresponds to the number of associated genes, and color intensity reflects the degree of enrichment. DPBS: Dulbecco’s phosphate-buffered saline; Exo: exosome.

To understand the functional implications of the DEGs between the Exo and DPBS groups, we performed GO enrichment analysis (**Figure 4**). The results highlighted that the DEGs were primarily associated with biological processes (BPs) related to immune response and immune system processes, as well as cellular components (CCs) associated with the membrane surface and intracellular components of host cells. In terms of molecular functions (MFs), the DEGs were mainly associated with chemokine receptor binding, chemokine activity, and cytokine activity. These findings suggested that exosome treatment modulates critical aspects of the immune and inflammatory responses, influencing CCs integral to these processes.

### KEGG pathway annotation and enrichment analysis of differentially expressed genes

**Figure 5:**
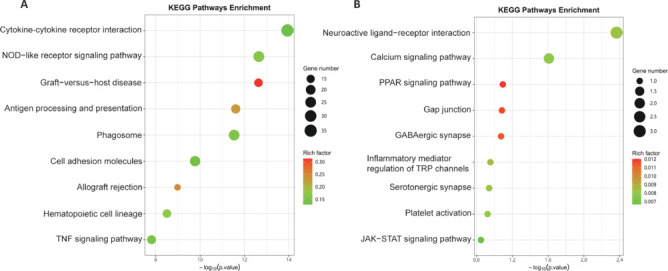
KEGG enrichment of DEGs in the Exo *vs*. DPBS group comparison. (A, B) KEGG pathways enriched with the upregulated DEGs (A) and downregulated DEGs (B) in the Exo group. The y-axes represent the names of enriched KEGG pathways and the x-axes represent the adjusted *P* value (–log10[*P* value]). The size of each point corresponds to the number of genes enriched in the pathway, while the color intensity reflects the degree of enrichment. DEGs: Differentially expressed genes; DPBS: Dulbecco’s phosphate-buffered saline; Exo: exosome; KEGG: Kyoto Encyclopedia of Genes and Genomes.

To identify the key signaling cascades modulated by exosome treatment, we performed KEGG pathway enrichment analysis on the DEGs between the Exo and DPBS groups. Upregulated DEGs in the Exo group primarily showed enrichment of pathways involving cytokine-cytokine receptor interaction signaling, graft-versus-host disease signaling, antigen processing and presentation signaling, transplant rejection signaling, cell adhesion molecule signaling, NOD-like receptor signaling, hematopoietic cell lineage signaling, and TNF signaling (**Figure 5A**). By contrast, the downregulated DEGs in the Exo group were mainly enriched in KEGG pathways involving the regulation of inflammatory mediators on transient receptor potential (TRP) channel signaling, calcium signaling, peroxisome proliferator-activated receptor (PPAR) signaling, gap junction signaling, GABAergic synapse signaling, serotonin synapse signaling, JAK-STAT signaling, and platelet activation signaling, among other pathways (**Figure 5B**). These results highlighted the broad impact of exosome treatment on a diverse range of signaling pathways, encompassing inflammation, immunity, and cellular communication, underscoring a complex and multifaceted mechanism of action.

### Validation of differential gene expression levels

**Figure 6:**
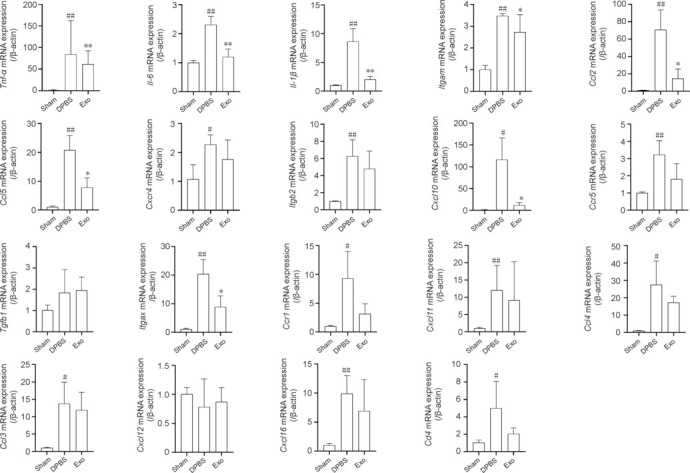
Effects of exosome treatment on mRNA expression of differentially expressed genes. Quantitative reverse transcription-polymerase chain reaction analysis of the mRNA expression levels of key differentially expressed genes identified in the transcriptomics analysis. mRNA levels were normalized to β-Actin expression. Data are presented as mean ± SEM (*n* = 3). #*P* < 0.05, ##*P* < 0.01, *vs.* Sham group; *P < 0.05, **P < 0.01, *vs*. DPBS group (one-way analysis of variance followed by Tukey’s *post hoc* test). Ccl11: Chemokine (C–C motif) ligand 11; Ccl12: chemokine (C–C motif) ligand 12; Ccl2: C–C motif chemokine ligand 2; Ccl3: chemokine (C–C motif) ligand 3; Ccl4: chemokine (C–C motif) ligand 4; Ccl5: C–C motif chemokine ligand 5; Ccr1: C–C chemokine receptor type 1; Ccr5: C–C chemokine receptor 5; Cd4: T-cell surface glycoprotein CD4; Cxcl10: C–X–C motif chemokine ligand 10; Cxcl16: C–X–C motif chemokine ligand 16; Cxcr4: C–X–C chemokine receptor type 4; Il-1β: interleukin-1β; Il-6: interleukin-6; Itgam: integrin subunit alpha M; Itgax: integrin subunit alpha X; Itgb2: integrin beta-2; Tgfb1: transforming growth factor beta 1; Tnf-α: tumor necrosis factor-α.

To validate the mRNA levels of key DEGs identified through transcriptomics, we performed qRT-PCR analysis of *Tnf-α*, *Il-6*, *Cd4*, *Il-1*β, *Itgam*, *Ccl2*, *Ccl5*, *Cxcr4*, *Itgb2*, *Cxcl10*, *Ccr5*, *Tgfb1*, *Itgax*, *Ccr1*, *Ccl11*, *Ccl4*, *Ccl3*, *Ccl12*, and *Cxcl16* in the Sham, DPBS, and Exo groups. As shown in **Figure 6**, the expression levels of *Ccl2*, *Ccl3*, *Ccl4*, *Ccl5*, *Ccl11*, *Ccr1*, *Ccr5*, *Cxcl10*, *Cxcl16*, *Cxcr4*, *Il-1*β, *Il-6*, *Itgam*, *Itgax*, *Itgb2*, *Cd4*, and *Tnf-α* were significantly upregulated in the DPBS group compared with those in the Sham group (*P* < 0.05). Compared with the DPBS group, MSC-derived exosome treatment in the Exo group led to significant downregulation of *Ccl2*, *Ccl5*, *Cxcl10*, *Il-1*β, *Il-6*, *Itgam*, *Itgax*, and *Tnf-α* (*P* < 0.05). The quantitative reverse transcription-polymerase chain reaction data largely corroborated the transcriptomic data, lending further support to the conclusion that exosome treatment effectively modulates the expression of key genes involved in inflammatory and immune responses following ischemic stroke.

### Differential metabolity analysis

**Figure 7:**
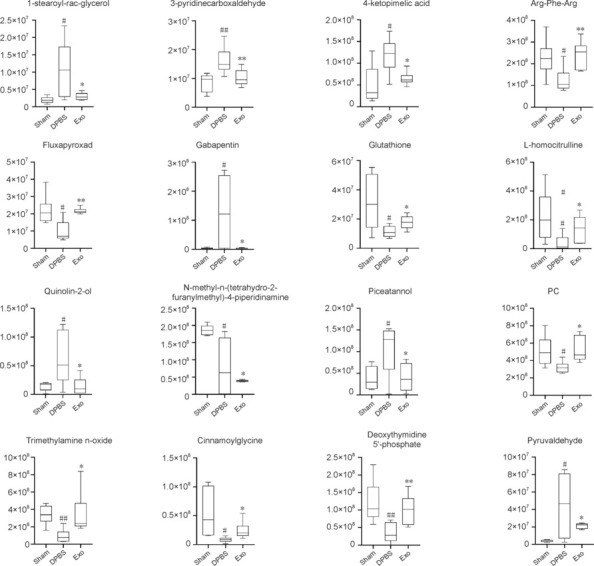
Differential metabolite analysis. Box plots showing changes in the levels of key metabolites. Data are presented as mean ± SEM with *n* = 6 per group. #*P* < 0.05, ##*P* < 0.01, *vs.* Sham group; *P < 0.05, **P < 0.01, *vs*. DPBS group (one-way analysis of variance followed by Tukey’s *post hoc* test). DPBS: Dulbecco’s phosphate-buffered saline; Exo: exosome; PC: phosphatidylcholine.

To identify key metabolic targets during MSC-derived exosome treatment of ischemic stroke, we performed DM analysis between the DPBS and Sham groups and the Exo and DPBS groups. Notably, exosome treatment in the Exo group reversed the changes in certain metabolites observed in the DPBS *versus* Sham group comparison. These metabolites included 1-stearoyl-rac-glycerol, 3-pyridinecarboxaldehyde, 4-ketopimelic acid, Arg-Phe-Arg, cinnamoylglycine, deoxythymidine 5′-phosphate (dTMP), fluxapyroxad, gabapentin, phosphatidylcholine, piceatannol, pyruvaldehyde, quinolin-2-ol, trimethylamine N-oxide (TMAO), glutathione (GSH), L-homocitrulline, and N-methyl-N-(tetrahydro-2-furanylmethyl)-4-piperidinamine. As shown in **Table 2** and **Figure 7**, these DMs may serve as key targets in MSC-derived exosome treatment for ischemic stroke.

**Table 2:** Potential metabolites of treatment for middle cerebral artery occlusion with MSC-derived Exo

Name	HMDB	Mode	DPBS *vs*. Sham	Exo *vs*. DPBS
1-Stearoyl-rac-glycerol	HMDB0258680	Positive	↓*	↓*
3-Pyridinecarboxaldehyde	HMDB0245978	Positive	↑**	↑**
4-Ketopimelic acid	HMDB0012266	Positive	↓*	↑*
Arg-Phe-Arg	HMDB0012985	Positive	↓*	↑**
Fluxapyroxad	HMDB0252068	Positive	↓*	↑**
Gabapentin	HMDB0005015	Positive	↑*	↓*
Glutathione	HMDB0003337	Positive	↓*	↑*
L-homocitrulline	HMDB0000679	Positive	↓*	↑*
N-acetyl-o-fluoro-dl-phenylalanine	HMDB0000512	Positive	↑*	↑**
N-methyl-n-(tetrahydro-2-furanylmethyl)-4-piperidinamine	HMDB0001189	Positive	↓*	↑*
PC	HMDB0000564	Positive	↓*	↑*
Piceatannol	HMDB0004215	Positive	↑*	↓*
Quinolin-2-ol	HMDB0247378	Positive	↑*	↓*
Trimethylamine n-oxide	HMDB0000925	Positive	↓**	↑*
2-Hydroxy-2-methylbutyric acid	HMDB0001987	Negative	↓*	↓*
Amobarbital	HMDB0015440	Negative	↑**	↑**
Cinnamoylglycine	HMDB0011621	Negative	↓*	↑*
Deoxythymidine 5&-phosphate (dTMP)	HMDB0001227	Negative	↓**	↑**
Harmol	HMDB0034217	Negative	↑*	↑**
Pyruvaldehyde	HMDB0001167	Negative	↑*	↓*

*P < 0.05, **P < 0.01. DPBS: Dulbecco’s phosphate-buffered saline; Exo: exosome.

### KEGG pathways enriched in differential metabolites between the Exo and DPBS groups

**Figure 8:**
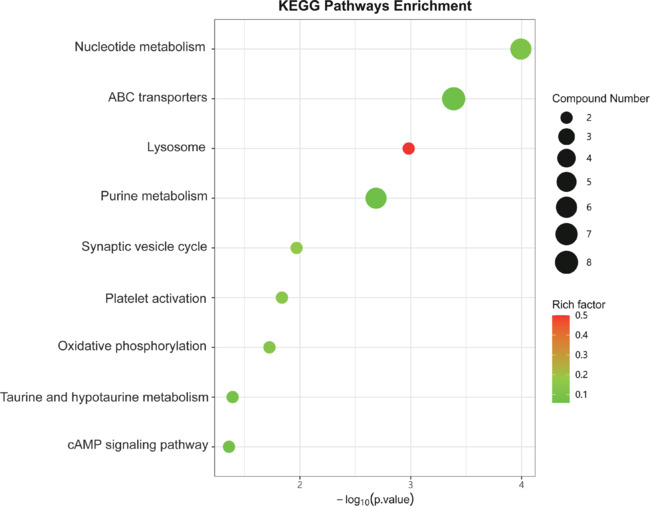
Kyoto Encyclopedia of Genes and Genomes (KEGG) pathway enrichment analysis of differential metabolites (DMs) between the Exo and DPBS groups. The *y*-axis represents the names of enriched KEGG pathways and the *x*-axis represents the adjusted *P* value (–log10[*P* value]). The size of each point is proportional to the number of DMs mapped to the corresponding pathway, while the color reflects the degree of enrichment. DPBS: Dulbecco’s phosphate-buffered saline; Exo: exosome.

To understand the functional implications of the observed metabolic changes, we performed KEGG pathway enrichment analysis of the DMs between the Exo and DPBS groups. As shown in **Figure 8**, the DMs were enriched in KEGG pathways primarily concentrated in nucleotide metabolism, ABC transporters, lysosomes, purine metabolism, the synaptic vesicle cycle, platelet activation, oxidative phosphorylation, taurine and hypotaurine metabolism, and cAMP signaling.

### Integrated transcriptomics and metabolomics analysis

**Figure 9:**
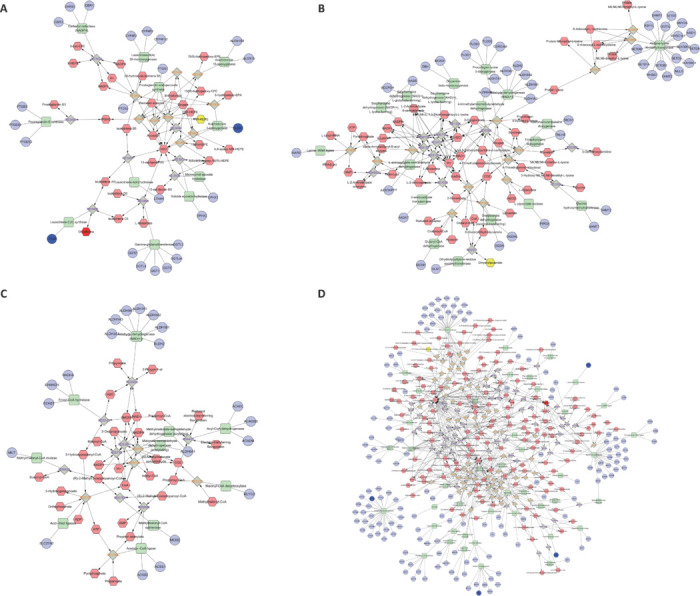
Network of compounds, reactions, and enzymes associated with the key metabolites and differentially expressed genes involved in the key metabolic pathways identified from integrated transcriptomics and metabolomics analysis. (A–D) Putative anti-inflammatory metabolites formed from metabolism of eicosapentaenoic acid (EPA; A), lysine (B), propanoate (C), and tyrosine (D). Compounds are indicated by hexagons, reactions by gray diamonds, proteins by green rounded rectangles, and genes by purple circles.

Next, we integrated the DM data from metabolomics and the DEG data from transcriptomics to identify significant metabolic pathways based on shared pathway patterns. To deepen our understanding of the mechanism of MSC-derived exosome therapy for ischemic stroke, we constructed a metabolite-protein-gene association network of the key metabolites and genes in these metabolic pathways. Metabolomic and transcriptomic correlation analysis was performed to elucidate the relationships between DMs and DEGs. Upstream and downstream genes regulating the production or degradation of DMs within these metabolic pathways were then identified using visualization and imaging tools in Cytoscape 3.7.2. Integration of transcriptomic and metabolomic data revealed four metabolic pathways significantly modulated by MSC-derived exosome therapy for ischemic stroke: eicosapentaenoic acid (EPA) metabolism (**Figure 9A**), lysine metabolism (**Figure 9B**), propanoate metabolism (**Figure 9C**), and tyrosine metabolism (**Figure 9D**). These findings highlighted the intricate interplay between gene expression and metabolic regulation in the therapeutic response to ischemic stroke.

## Discussion

Despite emerging evidence for the therapeutic efficacy of human UC-MSC-derived exosomes in ischemic stroke, the optimal timing for their administration remains largely uncharacterized. In this study using mice, early (6 hours post-ischemic stroke) exosome therapy resulted in significantly improved neurological function scores and cylinder test performance, as well as more significant reductions in infarct volume and hippocampal neuronal death, compared with late (3 days) therapy. We hypothesize that the superior efficacy of early exosome therapy in this context stems from its prompt, effective modulation of the inflammatory cascade at the ischemic site. Earlier intervention in the inflammatory process may reduce the local inflammatory response, leading to better therapeutic outcomes. Furthermore, we gained a comprehensive understanding of the therapeutic mechanisms underlying MSC-derived exosomes by conducting transcriptomic and metabolomic analyses of brain tissues from mice treated at the optimal (early) post-stroke time point.

Among the key DEGs identified through transcriptomic studies was *Ccl5*, one of several chemokine genes located on the q arm of chromosome 17 (Tian et al., 2022). Chemokines are a superfamily of secreted proteins that participate in immunoregulation and inflammatory processes. CCL5 acts as a chemoattractant for blood monocytes, memory T helper cells, and eosinophils. It also activates eosinophils, induces histamine release from basophils, and is involved in ischemic stroke pathology and monocyte recruitment (Wang et al., 2022). CCL5/CCR5 expression is reportedly increased in ischemic brain tissue, consistent with our experimental observations (Liu et al., 2023a). Inhibition of CCL5 reduces monocyte infiltration and alleviates monocyte-mediated inflammation in ischemic stroke. Here, the Exo group showed significant downregulation of *Ccl5* expression compared with the DPBS group, indicating that MSC-derived exosomes have a strong inhibitory effect on *Ccl5* expression. We speculate that MSC-derived exosomes reduce monocyte infiltration by suppressing *Ccl5* expression, thereby mitigating monocyte-mediated inflammatory responses.

*Cxcl10* encodes a key chemokine within the CXC subfamily (Bandow et al., 2022), serving as a ligand for the CXCR3 receptor. Binding of this protein to the CXCR3 receptor triggers a series of important biological effects, which include stimulating the migratory activities of monocytes, natural killer cells, and T cells, and finely regulating the expression of adhesion molecules. These findings have been corroborated by multiple studies (Guldner et al., 2020; Mikolajczyk et al., 2021; Piemonti et al., 2021; Lawrence et al., 2023), deepening our understanding of the role of *Cxcl10* in immunoregulation and cell migration. This is the first report of *Cxcl10* related to cerebral infarction. We hypothesize that MSC-derived exosomes achieve therapeutic effects by suppressing *Cxcl10* expression and ameliorating local inflammatory responses in cerebral infarction.

IL-1β, a member of the interleukin-1 cytokine family, is an important mediator in immune and inflammatory responses in a wide range of vertebrates. This cytokine, initially produced as a precursor protein by activated macrophages, is converted into its active form through cleavage by caspase-1 (CASP1/ICE) (Tang et al., 2023). IL-1β participates in regulating cellular activities essential for maintaining homeostasis and responding to environmental stimuli, such as proliferation, differentiation, and apoptosis (Zaben et al., 2021; Yanagihara et al., 2023). Within the central nervous system, this cytokine induces the expression of cyclooxygenase-2 (PTGS2/COX2). IL-1β-mediated inflammation can disrupt the blood–brain barrier, thereby facilitating the infiltration of immune cells into the central nervous system (Jagadapillai et al., 2022). It also leads to increased expression of chemokines, stimulates the migration of leukocytes into the brain parenchyma (Uddin et al., 2022), and induces the expression of Fas ligand on glial cells, exacerbating neuroinflammation and neuronal death (Higashikuni et al., 2023). When released in large amounts, IL-1β promotes the phosphorylation and activation of N-methyl-D-aspartate (NMDA) receptors, causing excessive calcium influx and subsequent excitotoxicity (Gaire, 2022). Our qRT-PCR analysis revealed that MSC-derived exosomes significantly suppressed the expression of IL-1β, leading us to speculate that MSC-derived exosomes reduce neuronal damage by lowering IL-1β levels.

The cytokine IL-6, encoded by a gene that plays roles in inflammation and B-cell maturation, is an endogenous pyrogen produced primarily at sites of acute and chronic inflammation. It is subsequently secreted into the serum, where it exerts biological functions involved in various inflammatory disease states (Williams et al., 2022). IL-6 expression is low in normal brain tissue, becoming significantly elevated during trauma, infection, stroke, and inflammation. During cerebral ischemia, IL-6 is produced by neurons, oligodendrocytes, astrocytes, and vascular endothelial cells. In ischemic stroke, microglia are rapidly activated, becoming key cells in the brain’s inflammatory response, secreting pro-inflammatory cytokines such as IL-6, IL-1β, and TNF-α, which contribute to microglia-mediated neurodamage (Guo et al., 2021). Previous research indicates that IL-6 levels increase during ischemic stroke (Zhang et al., 2019a). Some studies found that cytokines (e.g., TNF-α, IL-1α/β, and IL-6) affect phospholipid metabolism and produce eicosanoids, sphingolipids, and reactive oxygen species during acute inflammatory responses to brain ischemia, causing damage to brain tissue (Pu et al., 2022). Following vascular occlusion, increased expression of IL-1 and IL-6 affects vascular endothelial cells, prompting them to express ICAM-1, P-selectin, and E-selectin, leading to leukocyte aggregation and adhesion, and mediating an inflammatory cascade that exacerbates cerebral ischemic damage (Liberale et al., 2021). In our experiments, MSC-derived exosomes significantly suppressed IL-6 expression, thereby effectively inhibiting IL-6-mediated inflammatory responses.

*Itgax* encodes the integrin α X chain protein. Integrins are heterodimeric integral membrane proteins composed of α and β chains. When combined with the β2 chain (ITGB2), this protein forms a leukocyte-specific αXβ2 complex with properties similar to the αMβ2 integrin, which is involved in the adhesion of neutrophils and monocytes to stimulated endothelial cells and the phagocytosis of immune complexes (Zhang et al. 2019b). To our knowledge, this report is the first that links *Itgax* to stroke.

Our KEGG pathway enrichment analysis of DEGs in MCAO mice revealed that MSC-derived exosome treatment did not downregulate a specific signaling pathway; rather, it broadly downregulated inflammation-related signal pathways. These included pathways involving neuroactive ligand-receptor interaction, calcium signaling, JAK-STAT signaling, PPAR signaling, GABAergic synapses, modulation of TRP channels by inflammatory mediators, cytokine-cytokine receptor interaction, and other pathways. Given that current studies point to inflammation as a primary contributor to neuronal death in ischemic stroke (Przykaza, 2021), we hypothesize that early administration of MSC-derived exosomes exerts therapeutic effects by comprehensively downregulating inflammation-related signaling pathways, leading to improved neurological functional recovery. Furthermore, we discovered that MSC-derived exosomes also downregulate platelet activation. It is currently believed that, following brain infarction, endogenous neural repair activities are spontaneously initiated as an adaptive response to neuronal injury. These activities are usually observed on the fifth or fourteenth day after the onset of stroke (Lin et al., 2023), typically manifesting as brain microvessels and glial cells working together to create an environment conducive to neural repair. These brain microvessels accumulate in tissues around the ischemic site and extend to the damaged brain areas and other neurons, modulating synaptic activity and inducing the formation of new synapses. In our analysis of the DPBS and Exo groups, the downregulated DEGs in the Exo group were enriched in the platelet activation pathway, suggesting that treatment with MSC-derived exosomes downregulated this pathway. Importantly, our sampling time of 7 days post-cerebral infarction was consistent with reports on the timing of endogenous neural repair activities (Lin et al., 2023). We speculate that the application of MSC-derived exosomes downregulates the platelet activation pathway to create favorable conditions for such activities, aiding in the recovery of neural function.

Metabolomic screening of key metabolites involved in the treatment of ischemic stroke with MSC-derived exosomes included TMAO and GSH. TMAO is a byproduct of gut microbes closely associated with stroke. Current research on the interaction between TMAO and the nervous system are controversial, with some studies suggesting that serum TMAO has a protective effect, while others argue that serum TMAO is an indicator of disrupted homeostasis (Velasquez et al., 2016; Cho et al., 2017). In our analysis, compared with the Sham group, the level of TMAO was downregulated in the DPBS group but was upregulated in the Exo group. These findings suggested that TMAO plays a protective role, warranting further research on TMAO. GSH, a tripeptide composed of glutamate, cysteine, and glycine, is essential for maintaining physiological antioxidant functions. GSH has neuroprotective effects in various types of brain injury, including cerebral ischemia, hypoglycemia, and traumatic brain injuries (Iskusnykh et al., 2022). Stroke generates excess reactive oxygen species through complex biochemical cascades, exacerbating primary neuronal damage. GSH, by maintaining low reactive oxygen species levels, may improve cognitive function after stroke (Aoyama, 2021). In our experiments, GSH levels increased following treatment with MSC-derived exosomes, which may have contributed to the improvement in neural function.

Integrated transcriptomics and metabolomics analysis revealed a metabolite-protein-gene network, identifying four important metabolic pathways (EPA, lysine, propanoate, and tyrosine) correlated with DEGs. This led us to speculate that MSC-derived exosomes may influence these pathways to treat ischemic stroke in mice. Inflammation is thought to dominate the pathogenesis of ischemic stroke, during which production of inflammatory mediators, such as TNF, IL-6, and IL-2 increases, with arachidonic acid also playing a key role. EPA can be incorporated into platelet phospholipid membranes, reducing the synthesis of arachidonic acid derivatives and aggregation of platelets (Safouris et al., 2021). Although the link between the EPA metabolism pathway and ischemic stroke is not widely discussed in the literature, our findings lead us to speculate that this metabolic pathway is an important target for MSC-derived exosome therapy, warranting further research.

KEGG pathway enrichment analysis of the DMs between the Exo and DPBS groups highlighted pathways involving lysosomes, ABC transporters, nucleotide metabolism, thermogenesis, platelet activation, oxidative phosphorylation, taurine and hypotaurine metabolism, and the cAMP signaling pathway. Among these, we focused on the ABC transporters, which constitute an ancient and ubiquitous family of membrane proteins found in various organisms. These proteins translocate various bound substrates across membranes using the energy generated from ATP hydrolysis, playing a significant physiological role in both prokaryotes and higher eukaryotes (Nwabufo, 2022). The ABC transporter superfamily includes 48 members (Thomas and Tampé, 2020). Among these, ABCA1, ABCA3, ABCA4, ABCA7, ABCB1, ABCB4, and ABCC1 transport phospholipid substances across cellular membranes. ABCA1, exhibiting high expression in all types of brain cells, regulates various cellular functions, including phagocytosis, inflammation, intracellular signaling, vesicle recycling, and apoptosis. Mice lacking ABCA1 in the brain exhibit decreased phagocytic activity and increased brain levels of TNF-α, presenting an inflammatory phenotype (Bogie et al., 2020). Consistently, studies have reported brain inflammation in *ABCA1* knockout mice (Yalcinkaya et al. 2023). We speculate that ABC transporters also serve as a target for MSC-derived exosome treatment of ischemic stroke.

This study has some limitations. First, the scope of this research, while providing a systems-level view of the impact of exosomes on ischemic stroke in mice, did not allow for in-depth mechanistic investigation of specific signaling pathways. This multi-omics approach, while powerful for identifying broad trends, may have obscured nuanced effects of changes in specific genes and metabolites and their functional consequences. To address this possibility, our future work will focus on targeted mechanistic studies, validating key pathways implicated by our integrated transcriptomic and metabolomic analysis. This will involve *in vitro* and *in vivo* experiments designed to assess the functional consequences of differential gene and protein expression, using techniques such as gene editing, biochemical assays, and functional imaging.

In conclusion, our study provides compelling evidence for the superior therapeutic efficacy of early versus delayed administration of human UC-MSC-derived exosomes in ischemic stroke. The observed improvements in neurological function, reduced infarct volume, and diminished neuronal death can be attributed to a multifaceted mechanism involving the early and effective modulation of the inflammatory cascade at the ischemic site. Specifically, our omics approach has revealed coordinated downregulation of key pro-inflammatory genes and signaling pathways, alongside the modulation of crucial metabolites including TMAO and GSH, all of which converge to create an environment conducive to stroke recovery. Furthermore, downregulation of the platelet activation pathway suggests a potential for these exosomes to dampen the immediate inflammatory response while facilitating endogenous repair processes. Although these findings offer a valuable mechanistic framework, the intricate interplay between these factors warrants further investigation. Future studies should focus on dissecting the precise roles of the identified metabolic pathways, especially the EPA metabolism pathway and the involvement of ABC transporters, in the context of exosome-mediated neuroprotection. Additionally, longitudinal studies that track the dynamic changes in gene expression, metabolite levels, and neurobehavioral outcomes are crucial for fully realizing the therapeutic potential of UC-MSC-derived exosomes. Ultimately, a deeper understanding of these molecular mechanisms will pave the way for the development of more effective exosome-based therapies targeted at ischemic stroke, moving us closer to clinical translation.

## Data Availability

*No additional data are available*.
